# Quantitative differential proteomics of yeast extracellular matrix: there is more to it than meets the eye

**DOI:** 10.1186/s12866-015-0550-1

**Published:** 2015-11-25

**Authors:** Fábio Faria-Oliveira, Joana Carvalho, Célia Ferreira, Maria Luisa Hernáez, Concha Gil, Cândida Lucas

**Affiliations:** CBMA - Centro de Biologia Molecular e Ambiental, Departamento de Biologia, Universidade do Minho, Campus de Gualtar, 4710-057 Braga, Portugal; Unidad de Proteómica, Universidad Complutense de Madrid – Parque Científico de Madrid (UCM-PCM), Madrid, Spain; Departamento de Microbiología II, Facultad de Farmacia, Universidad Complutense de Madrid, Madrid, Spain

**Keywords:** *Saccharomyces cerevisiae*, Extracellular matrix, ECM, Proteome, Nano LC-MS/MS, DIGE, Metalloproteinases, Chaperones, Extracellular signals

## Abstract

**Background:**

*Saccharomyces cerevisiae* multicellular communities are sustained by a scaffolding extracellular matrix, which provides spatial organization, and nutrient and water availability, and ensures group survival. According to this tissue-like biology, the yeast extracellular matrix (yECM) is analogous to the higher Eukaryotes counterpart for its polysaccharide and proteinaceous nature. Few works focused on yeast biofilms, identifying the flocculin Flo11 and several members of the HSP70 in the extracellular space. Molecular composition of the yECM, is therefore mostly unknown. The homologue of yeast Gup1 protein in high Eukaryotes (*HHATL*) acts as a regulator of Hedgehog signal secretion, therefore interfering in morphogenesis and cell-cell communication through the ECM, which mediates but is also regulated by this signalling pathway. In yeast, the deletion of *GUP1* was associated with a vast number of diverse phenotypes including the cellular differentiation that accompanies biofilm formation.

**Methods:**

S. cerevisiae W303-1A wt strain and *gup1∆* mutant were used as previously described to generate biofilm-like mats in YPDa from which the yECM proteome was extracted. The proteome from extracellular medium from batch liquid growing cultures was used as control for yECM-only secreted proteins. Proteins were separated by SDS-PAGE and 2DE. Identification was performed by HPLC, LC-MS/MS and MALDI-TOF/TOF. The protein expression comparison between the two strains was done by DIGE, and analysed by DeCyder Extended Data Analysis that included Principal Component Analysis and Hierarchical Cluster Analysis.

**Results:**

The proteome of *S. cerevisiae* yECM from biofilm-like mats was purified and analysed by Nano LC-MS/MS, 2D Difference Gel Electrophoresis (DIGE), and MALDI-TOF/TOF. Two strains were compared, wild type and the mutant defective in *GUP1.* As controls for the identification of the yECM-only proteins, the proteome from liquid batch cultures was also identified. Proteins were grouped into distinct functional classes, mostly *Metabolism, Protein Fate/Remodelling* and *Cell Rescue and Defence* mechanisms, standing out the presence of heat shock chaperones, metalloproteinases, broad signalling cross-talkers and other putative signalling proteins. The data has been deposited to the ProteomeXchange with identifier PXD001133.

**Conclusions:**

yECM, as the mammalian counterpart, emerges as highly proteinaceous. As in higher Eukaryotes ECM, numerous proteins that could allow dynamic remodelling, and signalling events to occur in/and via yECM were identified. Importantly, large sets of enzymes encompassing full antagonistic metabolic pathways, suggest that mats develop into two metabolically distinct populations, suggesting that either extensive *moonlighting* or actual metabolism occurs extracellularly. The *gup1∆* showed abnormally loose ECM texture. Accordingly, the correspondent differences in proteome unveiled acetic and citric acid producing enzymes as putative players in structural integrity maintenance.

## Background

The yeast *Saccharomyces cerevisiae* is the most studied lower Eukaryote. As all microorganisms, it is predominantly regarded as a unicellular organism. Yet, *S. cerevisiae*, as any microbe, can form large multicellular communities: biofilms, colonies and stalks [1]. All these are formed by extremely large numbers of cells, sustained by a scaffolding extracellular polymeric substance (EPS), forming a network of channels conducting water and nutrients to the cells farther from the surrounding medium [2–5]. This is accompanied by large differences in gene expression and metabolic performance between cells in different layers [4, 6–8], with consequences at the level of biological processes [5–8]. Accordingly, active roles of the EPS from colonies and biofilms in the protection against xenobiotics and desiccation have been described in *S. cerevisiae* [5, 6] and *C. albicans* [9]. These observations encompass a new conceptualization of microbial life, taking colonies and other large aggregates of cells as the simplest forms of multicellular organization, with tissue-like biology, ensuring spatial organization and group survival. The molecular characterization of the yeast EPS, herein designated by yeast extracellular matrix - yECM - is still incipient. *S. cerevisiae* colonies ECM displays large amounts of glycoproteins [3], namely the flocculin Flo11 [7], while ECM from *C. albicans* biofilms has been reported to contain proteins, sugars and DNA [10, 11]. In this last case, several proteins from carbon metabolism were identified, namely several glycolytic and fermentative enzymes, as well as members of the HSP70 family [12, 13].

The yeast proteins Gup1 and Gup2 are highly conserved in all Eukaryotes [14, 15], namely in mammals, rodents [14, 16] and humans [17]. Otherwise, the fly [18] and the nematode [19] have only one Gup2 orthologue, while several fungi [20], and the yeast *C. albicans* [21], present only one Gup1 orthologue. Both Gup1 and Gup2 proteins belong to the MBOAT Membrane Bound *O*-Acyltransferases family, although neither displays the correspondent enzymatic activity [22]. In spite of the absence of a recognizable biochemical role, these proteins, in particular Gup1, were associated in high Eukaryotes with a modification of the Hedgehog morphogen that precludes its export from the cell [14]. For this reason, in all non-yeast organisms, Gup1 is known as HHATL (Hedgehog Acyl Transferase-Like protein) [14], and Gup2 as HHAT (Hedgehog Acyl Transferase). In mammals, ECM is the major vehicle and modulator of Hedgehog signalling, to which it responds with ever changing molecular structure and composition [23–25]. Hedgehog pathway is one of the most important signalling pathways from high Eukaryotes, promoting long distance cell-cell communication through the ECM [26], commanding crucial events during embryogenesis and wound healing, namely cellular differentiation, patterning and migration [27]. ECM therefore controls the Hh signal diffusion [28, 29] and at the same time has its composition controlled by Hh pathway [30]. In yeast communities, neither a diffusible cell-to-cell communication signal has been identified, nor an Hh-like pathway was described. In *S. cerevisiae* and *C. albicans,* the deletion of *GUP1* has been associated with a vast number of phenotypes from major biological processes, namely plasma membrane and cell wall molecular composition, biogenesis and structure [31, 32], endocytosis and cytoskeleton organization [33, 34], differentiation into hyphae and budding patterning [35, 36]. The influence on Gup1 in the production of yECM and the correspondent proteome were therefore assessed using *S. cerevisiae gup1∆* and wt strains.

The proteins secreted into the yECM from a homogenous overlay/mat [37] were identified by quantitative proteomic analysis, 2D Difference Gel Electrophoresis (DIGE), and compared with the proteins identified on the liquid growth media as control for yECM-only proteins. This work presents the first comprehensive analysis of yeast extracellular matrix proteome. yECM emerges as a highly proteinaceous environment, displaying multiple chaperones and metalloproteinases, and several broad signalling cross-talkers and putative signalling proteins. This suggests that analogously to higher Eukaryotes ECM, remodelling and signalling events occur in yECM. Furthermore, large sets of enzymes encompassing full antagonistic metabolic pathways, suggest that mats develop into two metabolically distinct populations, and also that extracellular metabolism might occur. Additionally, the presence of so many enzymes outside the cell might also encompass extensive *moonlighting*. The comparison between the *S. cerevisiae* wt strain and the mutant defective in the *GUP1* gene displaying abnormal ECM texture, was fruitful in which it contributed to recognize yECM composition is dynamically regulated and allowed to unveil putative players in structural integrity maintenance.

## Results and discussion

**Fig. 1:**
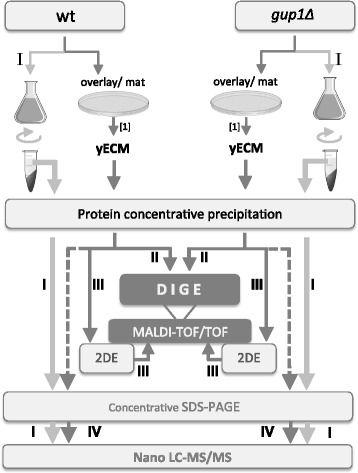
Experimental strategy used to identify the major protein players from yECM in *S. cerevisiae* wt and *gup1∆* mutant. Experimental paths I – IV

The overall strategy of the present work is presented in Fig. 1. In order to produce *S. cerevisiae* ECM, a biofilm-like overlay/mat of cells was cultured as previously described [37]. This allows the recovery of large amounts of yECM that are not possible to obtain from single colonies or biofilms. The cells in the mat were separated from the surrounding yECM, and this was fractioned into analytical-grade protein and sugar fractions [37]. To pinpoint the proteins that are particular to yECM, the proteins secreted into liquid growth medium (LGM) , from batch cultures were equally identified (Fig. 1 - path I). The protein fractions from both yECM and LGM were obtained by precipitation using a protocol known for its high performance in removing salts and detergents [38]. The use of this protocol was particularly important to avoid interference of buffer salts in the downstream analysis by polyacrylamide gel-based electrophoresis (2DE and SDS-PAGE), and mass spectrometry through MALDI-TOF/TOF and Nano LC-MS/MS.

Two *S. cerevisiae* strains were chosen for comparison, a wild type strain and a correspondent mutant strain defective for *GUP1* gene in view of its role in higher Eukaryotes in Hedgehog pathway signalling and consequent indirect ECM composition regulation. Compared to wild type strain, the mats developed by *gup1∆* presented a differently textured soft jelly-like yECM (not shown). The comparison of yECM proteome from *gup1∆* mutant and wt should therefore indicate candidate proteins involved in yECM physicochemical and molecular properties and functions, while the comparison with the proteome secreted into LGM by either strain should enable the identification of proteins specific from yECM.

The yECM from both wt and *gup1∆* mutant strains was extracted and the protein fraction identically purified and analysed (Fig. 1). Results were compared by 2D Difference Gel Electrophoresis (DIGE), followed by identification of proteins by MALDI-TOF/TOF (Fig. 1 – path II). Alternatively, the same samples were subjected to protein separation by 2DE and spots were identified by MALDI-TOF/TOF (Fig. 1 – path III), and concentrated by SDS-PAGE with subsequent identification of proteins by Nano LC-MS/MS (Fig. 1 – path IV). The same procedures were used to identify the proteins secreted into LGM (Fig. 1 – path I).

### Compared analysis of *S. cerevisiae* wt and *∆gup1* ECM proteome by Nano LC-MS/MS

#### Global results

**Fig. 2:**
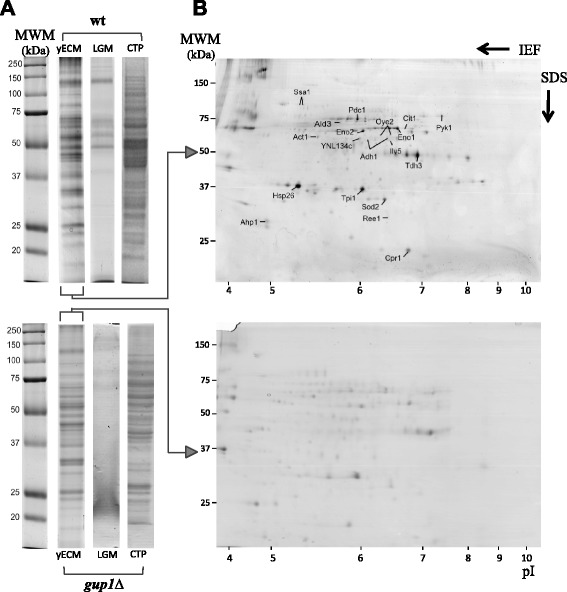
Analysis of the total yECM proteome from *S. cerevisiae* wt and *gup1∆* mutant. **a** SDS-PAGE of yECM is compared with cell’s total proteome (CTP) from cells grown in solid media and liquid growth medium (LGM). Each lane displays a different pattern though some bands are clearly common. **b** 2DE analysis of wt yECM protein extract in lane 2. The protein in marked spots were identified by MALDI-TOF/TOF: Act1 (actin 1), Adh1 (alcohol dehydrogenase 1), Ahp1 (alkyl hiydroperoxide reductase 1), Ald3 (aldehyde dehydrogenase 3), Cit1 (citrate synthase 1), Cpr1 (cyclosporin A-sensitive proline rotamase 1), Eno1 (enolase 1), Eno2 (enolase 2), Hsp26 (heat shock protein 26), Ilv5 (isoleucine-plus-valine requiring enzyme 5), Oye2 (old yellow enzyme 2), Pdc1 (pyruvate decarboxylase 1), Pyk1 (pyruvate kinase 1), Ree1 (regulation of enolase 1), Sod2 (superoxide dismutase 2), Ssa1 stress-seventy subfamily A 1), Tdh3 (triose-phosphate dehydrogenase 3), Tpi1 (triose-phosphate isomerase), and the uncharacterized ORF *YNL134C*

The range of protein separation obtained by a 2DE was 17–120 kDa and 4–10 pI (Fig. 1 – path III). This was complemented applying Nano LC-MS/MS to a sample concentrated by SDS-PAGE (Fig. 1 – path IV), allowing the separation of proteins from 3–430 kDa and 3.88-11.36 pI [39]. These extracts were compared with the proteins secreted by liquid batch cultures, LGM, for Liquid Growth Medium, and with the whole cells proteome, TCP, for Total Cell Proteome, displaying a clearly different pattern of bands (Fig. 2a). As previously described [32], the wt strain LGM sample showed an extracellular protein profile less abundant than *gup1∆* mutant. These samples were subjected to Nano LC-MS/MS (Fig. 1 – path I), which identified respectively 80 and 313 proteins. Furthermore, the yECM proteins from both strains were separated by 2DE. Fig. 2b is representative of 4 identical independent assays per strain. The identification by MALDI-TOF/TOF of the most prominent spots from Fig. 2b yielded the proteins listed in Tables 1 and 2. All these proteins were also present in the sample analysed by Nano LC-MS/MS (Fig. 1 – path IV), but this technique allowed the further unequivocal identification of 694 yECM proteins from *S. cerevisiae* wt and 587 from the *gup1∆* mutant. The full lists of yECM and LGM proteins from both strains were deposited in the ProteomeXchange database - identifier PXD001133.

**Table 1:** Proteins identified applying MALDI-TOF/TOF to the most prominent spots from 2DE

*S. cerevisiae* W303 (Wt)	
Act1	Actin 1
Adh1	Alcohol dehydrogenase 1
Ahp1	Alkyl hydroperoxide reductase 1
Ald3	Aldehyde dehydrogenase 3
Cit1	Citrate synthase 1
Cpr1	Cyclosporin A-sensitive proline rotamase 1
Eno1	Enolase 1
Eno2	Enolase 2
Hsp26	Heat shock protein 26
Ilv5	Isoleucine-plus-valine requiring enzyme 5
Oye2	Old yellow enzyme 2
Pdc1	Pyruvate decarboxylase 1
Pyk1	Pyruvate kinase 1
Ree1	Regulation of Enolase 1
Sod2	Superoxide dismutase 2
Ssa1	Stress-seventy subfamily A 1
Tdh3	Triose-phosphate dehydrogenase 3
Tpi1	Triose-phosphate isomerase
*YNL134C*	Uncharacterized ORF

**Table 2:**
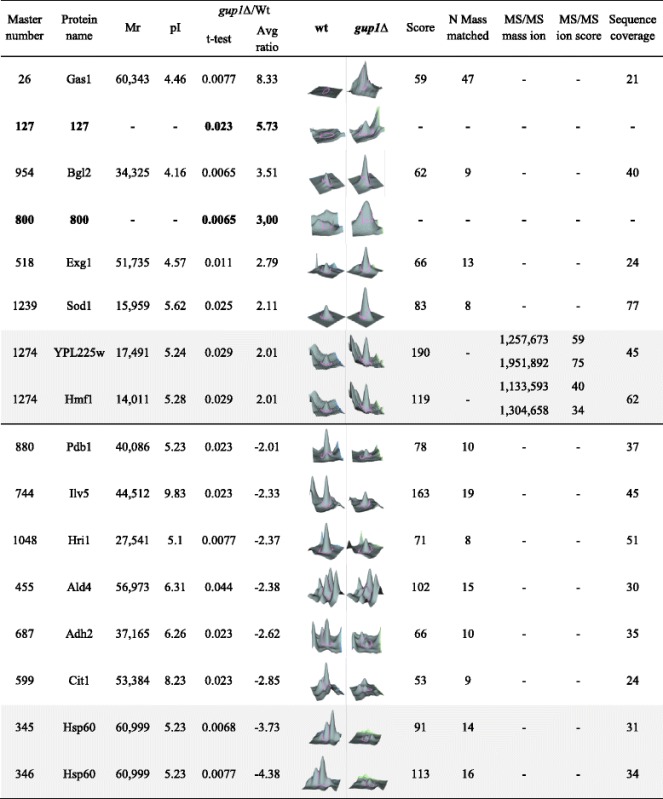
DIGE results: proteins identified in different amounts in the *S. cerevisiae* wt and *gup1∆* mutant yECM. Statistical analysis. Identification data and 3D representation of the spots are presented

Shadowed rows: upper half shows 2 proteins (each with 2 fragments identified totalling the coverage mentioned), lower half shows two spots identified as the same protein. Bold: proteins that were not identified due to low amount. Proteins listed abundance varied 2-fold or more between the wt and *gup1∆* yECM, and presented *p* < 0.05

#### *Functional distribution of proteins from* S. cerevisiae *wt ECM onto relevant families*

**Fig. 3:**
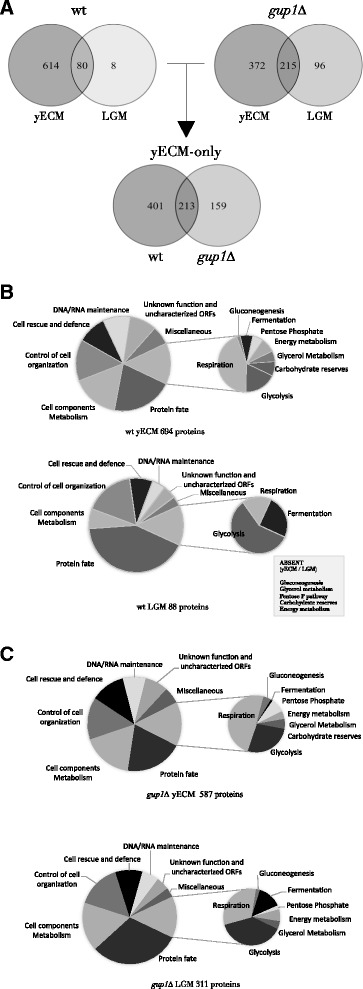
Global analysis of the proteins identified by LC-MS/MS in the yECM and LGM of wt and *gup1∆* mutant strains. **a** Numbers of proteins unequivocally identified by GC-MS/MS in yECM and liquid growth medium LGM, of *S. cerevisiae* wt and *gup1∆* mutant: 401 proteins are exclusive of wt yECM, 213 of which are common to *gup1∆* mutant, while 159 are exclusive of this mutant yECM. In opposition, and as previously reported [26], the *gup1∆* mutant secretes 3.5x more proteins into liquid growth medium than wt strain. **b** Compared functional distribution of the proteins found by LC-MS/MS in *S. cerevisiae* wt yECM (*upper panel*) and in liquid medium LGM (*lower panel*). Functional classes absent from LGM are mentioned in grey insert. **c** Identical compared functional distribution from the proteins found in *gup1∆* yECM (*upper panel*) and in LGM (*lower panel*)

From the *S. cerevisiae* wt ECM 694 proteins (db PXD001133), only 62 are presently uncharacterized ORFs or have no attributed function. The remaining 630 have known or predicted roles spread by a wide range of functional groups, from which *Metabolism*, broadly taken, and *Protein Fate/Remodelling* account for 51 % of the yECM proteins, while ≈ 10 % are proteins involved in *Cell Rescue and Defence* mechanisms (Fig. 3b, c), which includes signalling proteins.

#### Metabolism

The group of Carbon Metabolism includes all the enzymes necessary to accomplish glycolysis, alcoholic fermentation, as well as gluconeogenesis, namely Fbp1 fructose-1,6-bisphosphatase, and Pyc2 pyruvate carboxylase (db PXD001133). Furthermore, the glyoxylate and/or TCA cycles, aconitase Aco1, citrate synthase Cit1, malate synthase Mls1 and dehydrogenase Mdh1, isocitrate liase Icl2, and isocitrate dehydrogenases Idh1/2/ and Idp2, were also found (db PXD001133). Actually, from the 12 top scored proteins (scoring from 5000 to 10,000), 8 belong to these groups: Eno1 and Eno2, Tdh3, Fba1, Pgk1, Tdh1, Pyk1 and Pdc1. Enolase 1 (Eno1) and glyceraldehyde-3-phosphate dehydrogenase (Tdh1) are well known for targeting the cell surface of *C. albicans* [13]*,* and being secreted to the extracellular medium through unconventional means in *S. cerevisae* [40]. Eno1 also exhists in animal cells surface where it acts as a plasminogen receptor [41].

Besides glycolysis/gluconeogenesis, also the metabolism of amino acids, nucleotides and lipids were represented. yECM therefore displays large sets of enzymes from both glycolytic and gluconeogenic pathways. The presence of proteins which genes are known to be repressed by glucose, like Pck1, Mls1 and Icl2, suggests that yeast cells might have developed into two metabolically distinct populations, one under glucose repression, using glucose available directly from the growth medium, and another derepressed, putatively feeding on the ethanol generated by the first population. In colonies of *S. cerevisiae*, there has been reference to the existence of two metabolically diverse sub populations promoting nutrients flow inside the colony [42, 43]. Identically, *C. albicans* biofilms display sub-populations of morphologically and metabolically distinct cells [44]. In liquid grown yeasts, where there is unrestricted access to nutrients, only few of these glycolytic enzymes were previously reported at the cell surface [39, 45, 46]. Accordingly, in LGM samples these proteins were absent (db PXD001133).

#### Protein fate/remodelling

**Fig. 4:**
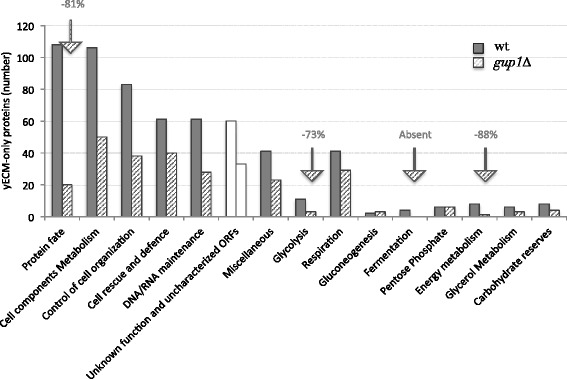
Compared functional distribution of the yECM-only proteins in wt and *gup1∆* mutant strains. *S. cerevisiae* wt (dark grey bars), *gup1∆* mutant (dashed bars), unknown/uncharacterised ORFs (white bars). Arrows indicate the highest percentage of reduction in numbers of proteins in mutant ECM compared to wt’s

From all the proteins found in *S. cerevisiae* ECM (db PXD001133), 150 are involved in the synthesis, folding and degradation of other proteins (Fig. 4). These include the proteins from the HSP70 family, Ssa1/2/3/4, Ssb1, Ssc1, Sse1/2 and Kar2, and the proteases, Lap4, Dug1, Ecm14, Ape2, Prd1 and Zps1. The HSP70 are chaperones responsible for the folding and membrane translocation of other proteins [47]. In particular, Ssa1 and Ssa2 are implicated in the biosynthesis and assembly of the cell wall [48]. These two proteins, as well as Ssb2 and Sse1 were previously reported to be present in the cell surface in both *S. cerevisiae* and *C. albicans* [39, 45, 46], and were found in this work in the top 12 most scored proteins in yECM above mentioned (db PXD001133). Moreover, also a number of proteins performing roles of cellular organization were identified. The wt yECM samples present several proteins involved in the cytoskeleton organization, namely the tubulin Tub2, the Arc19, Arc35, Arp2, Ent2 and Ent3 proteins have a role in the assembly of actin cortical patches, and the Rvs161 and Vps1 modulate the cytoskeleton assembly.

In mammalian ECM, metalloproteinases ensure constant remodelling of the glycoproteins [49]. Yeasts do not have a group of recognized metalloproteinases. Nevertheless, from the proteases found in *S. cerevisiae* yECM, Lap4 is a Zn-metalloproteinase [50], Dug1 is a metallodipeptidase [51], Ecm14 is a Zn-carboxipeptidase [52], Prd1 is a metalloendopeptidase [53], and Ape2 is an aminopeptidase [54]. Lap4, Dug1 and Ecm14 share the functional domain Zn–dependent exopeptidase, therefore belonging to the same Zn-dependent exopeptidases superfamily 53187, which includes another 11 yeast members not found in this work. On the other hand, Prd1 and Ape2 have a functional *zincin* domain. This domain is also present in another protein identified in yECM with unknown function, Zps1. Prd1, Ape2 and Zps1, together with 6 other yeast proteins not found in the present survey of yECM, belong to the metalloproteases *zincins* superfamily 55486, that includes, importantly, mammalian ECM metalloproteinases [55]. In higher Eukaryotes, ECM demands for constant remodelling. Analogously, we suggest that in *S. cerevisiae* ECM such a function might be performed by the hereby-identified metalloproteinases Lap4, Dug1 and Ecm14 from the Zn-dependent exopeptidases superfamily, and Prd1, Ape2 and Zps1 from the metalloproteases *zincins* superfamily, as well as the HSP70 chaperones Ssa1/2/3/4, Ssb1, Ssc1, Sse1/2 and Kar2. From these proteins, only Lap4, Ecm14, Zps1, Ssc1 and Sse2 were not found in the *S. cerevisiae* extracellular vesicles proteome [40, 56]. The finding that yeasts secrete vesicles containing large amounts of supposedly intracellular proteins comes in agreement with the present results and suggest that this could also be a mechanism underlying yECM proteome formation.

#### Cell rescue and defence - signalling

The group of proteins that were ascertained to the broad designation of *Cell Rescue and Defence* includes proteins involved in several stress responses, signalling processes and pH homeostasis (db PXD001133). In particular, Bmh1 and Bmh2 also known as 14-3-3 proteins were found. These are important proteins transversal to a large number of signalling pathways in mammalian cells, including TOR pathway and retrograde response, HOG, PKC and Ras/cAMP pathways, and chitin synthesis control [57, 58]. Both proteins were previously observed in the cell surface of both *S. cerevisiae* and *C. albicans* [39, 45, 59]. The presence of such broad signalling cross talkers in the yECM suggests that, identically to higher Eukaryotes, signalling events occur through the matrix, therefore implying cell-to-cell communication. In the same trend, the subunit A of the V1 domain of the vacuolar ATPase Tfp1 stood out. Tfp1 is the designation of the ORF that actually encodes for two proteins, the vacuolar membrane ATPase Vma1, and the *intein* homing endonuclease Vde, also known as PI-SceI [60]. In the present survey, the peptides that originated the identification of Tfp1 (db PXD001133) correspond to three different parts of this ORF, two from the Vma1 sequence in the beginning and in the end of the protein, and the other inside the Vde sequence. This means that the yECM may actually harbour either or all of Tfp1, Vma1 and Vde [60]. Western Blot using a rabbit anti-Vde antibody was done to confirm the presence of Vde. This antibody is capable of recognizing Vde before and after the auto-excision process, yielding several molecular size bands. WB was performed against the protein extracts from i) total cells (including all the intracellular proteins), ii) yECM, and iii) LGM (not shown). A prominent 50 kDa band from Vde was found, as well as much lower amounts of the higher and lower molecular weight intermediates of 90 kDa, 80 kDa, 40 kDa and 30 kDa, which should correspond to, respectively, Vde/Vma1-C’, Vde/Vma1-N’, Vma1-C’and Vma1-N’ [60]. In all cases, the bands indicated less protein in yECM than in total cell protein extracts and residual amounts in LGM (not shown). Although these results indicate that Vde peptides are in much higher quantity inside the cells than outside, they also show that their amounts in liquid media are almost inexistent for which their presence in yECM might be significant.

#### Proteins differentially expressed in wt and gup1∆ strains ECM

As mentioned above, using Nano LC-MS/MS, yECM extracts from *gup1∆* yielded 587 proteins (db PXD001133), 15 % less than wt. Still, the two strains shared 35 % of the total yECM proteome (213 proteins). In opposition, *gup1∆* LGM yielded 311 (three times more proteins than wt), 215 of which common to the mutant yECM (Fig. 3a; db PXD001133). Considering both score value and whole protein sequence coverage as indirect and approximate indications of the protein representation in the sample, the top 52 and 76 proteins scored more than 1.000 in the wt and *gup1∆* ECM samples respectively, while in liquid medium, only 3 of the 88 proteins, and 17 out of 311 scored that high (db PXD001133). This shows that ECM, regardless to the strain, is richer in proteins than the LGM counterpart, and that the deletion of *GUP1* selectively interferes with protein availability in the extracellular space both in solid and liquid growth conditions.

The *gup1Δ* LGM samples presented a great number of cell surface proteins, including cell wall and plasma membrane proteins (db PXD001133). These include plasma membrane integral proteins as Pma1 and GPI-anchored proteins, namely several members of the Gas family that are crucial for the cell wall remodelling, Gas1, Gas3 and Gas5. Gas1 was already identified as one of the proteins the mutant secretes into LGM more abundantly than wt strain [32]. The abnormally high release to the extracellular medium of the GAS proteins, as well as Pir1, Pir2 and Pir3 required for cell wall organization and maintenance, agrees with the compromised cell wall and membrane composition and structure previously reported for the *gup1Δ* mutant cells [31, 32, 61, 62]. Moreover, the *gup1Δ* LGM samples also presented a high number of proteins intervening in the carbon metabolism (db PXD001133), namely Eno1 (enolase), Fbp1 (fructose-1,6-bisphosphatase), and Tpi1 (triosephosphate isomerase). These proteins were already reported in the cell surface of other yeast strains during liquid growth [45, 59], but never before in the growth medium. Concurrently, they were not found in LGM from the wt. Additionally, also proteins associated with lipid synthesis (Plb family), ergosterol (Erg family), and fatty acids metabolism (Fas family), were present in the *gup1Δ* mutant sample (db PXD001133).

In opposition to LGM, mutant cells secreted into the yECM 15 % less proteins than wt (Fig. 3a), a large number of which were common to wt yECM (Fig. 3a, c) , including a great number of proteins involved in the cell wall remodelling that were not found in the wt yECM (Fig. 3c). Notably, the homologous GPI-anchored putative mannosidases Dcw1 and Dfg1, required for cell wall biosynthesis, the Utr2, Kre6 and Krt2 proteins involved in the biosynthesis of β-glucans, the Pir1 and Pir2 proteins involved in the stabilization of the cell wall, and the GPI-anchored protease Yps1 required for the cell wall growth and maintenance were found (db PXD001133). Additionally, the selective presence in the wild type ECM of alpha-saccharides remodelling enzymes, such as the Glc3 1,4-alpha-glucan branching enzyme, the Mnn2 alpha-1,2-mannosyltransferase, and the Sga1 alpha-glucoamylase, suggest the presence of alpha-based polysaccharides in opposition to the cell wall beta sugars’ backbone, and that these might be important for ECM texture as inferred from the absence of these enzymes from the *gup1∆* mutant ECM.

Globally, the proteins found in yECM of both strains belong to very diverse pathways, mechanisms and structures (Fig. 3b, c). The 4 of the top 5 scoring proteins of wt belong to glycolysis: enolase isoenzymes Eno1 and Eno2 already identified in wt ECM by 2D electrophoresis (Fig. 2b), glyceraldehyde-3-phosphate dehydrogenase Tdh1, and fructose 1,6-bisphosphate aldolase Fba1. The 5^th^ protein was the putative glucanase Scw4. Extracting the proteins common to yECM and LGM in either strain, yECM-only proteins for either strain were identified (Fig. 4). Approximately 50 % of these are common to wt and mutant strains (db PXD001133). The remaining proteins belong to the same functional categories, being the most affected functional groups the ones including proteins associated with cellular components and the metabolism of carbon and energy (Fig. 4). In particular, in the mutant yECM, no fermentation or gluconeogenic enzymes were found, as well as proteins that regulate this last pathway, namely the Fbp1 (fructose-1,6-bisphosphatase). Fbp1 is under tight glucose regulation [63]. A recent report [64] showed that the addition of glucose to starved cells lead to the degradation of both intra and extracellular Fbp1, suggesting that the role of the protein once in the cell surface is also glucose-dependent. Nevertheless, the results in the present work suggest otherwise, since the absence of Fbp1 from *gup1∆* mutant yECM is accompanied by the presence of other glucose-repressed proteins, Pck1 (phosphoenolpyruvate carboxykinase) and Mls1 (malate synthase).

The differences in protein identified in the wt and the *gup1∆* mutant suggest that the mutant could not form two metabolically distinct subpopulations, one fermenting the glucose present in the medium and the other respiring the ethanol and/or glycerol produced by the first. This is corroborated by the further absence from the mutant yECM of the pyruvate decarboxylase isoforms Pdc5 and Pdc6, while these and Pdc1 isoforms were all detected in the wt yECM. Pdc5 and Pdc6 are enzymes involved in glucose fermentation into ethanol, which expression is repressed by Pdc1 [65, 66].

### Compared analysis of wt and *∆gup1* yECM protein abundance by DIGE

#### Global results

Besides the differences in the absence/presence of proteins, the yECM from the wt and *gup1∆* mutant also differ in the expression of other proteins. In order to deeper characterize these differences, the yECM protein samples of both wt and *gup1∆* strains were subjected to two-dimensional differential gel electrophoresis (DIGE) (Fig. 1 - path II). This highly sensitive technique allowed the direct comparison of samples from both strains in a same gel, eliminating the inter-gel comparison bias. A crucial characteristic of this technique is the presence of the internal standard labelled with Cy2 dye, which is formed by equal amounts of all samples, and it is included in all gels, allowing the normalization of measured fluorescence. In order to avoid dye-dependent variations, namely labelling efficiency and fluorescence emission, half of the samples from each strain yECM protein fraction were labelled with Cy3 dye, and the other half was labelled with Cy5 dye (Table 3). This allowed to eliminate most of the inherent variation between the gels and enabled a statistically validated analysis with the four gels acting as independent replicates.

**Table 3:** Scheme of CyDye labelling used in Wt/*gup1∆* DIGE. Four samples, originating from independent cell cultures of each strain, were used for differential labelling as indicated

Gel/Dye combination	Cy5	Cy3	Cy2
I	Wt (1)	*gup1Δ* (4)	Internal standard
II	Wt (2)	*gup1Δ* (1)	Internal standard
III	*gup1Δ* (2)	Wt (3)	Internal standard
IV	*gup1Δ* (3)	Wt (4)	Internal standard

**Fig. 5:**
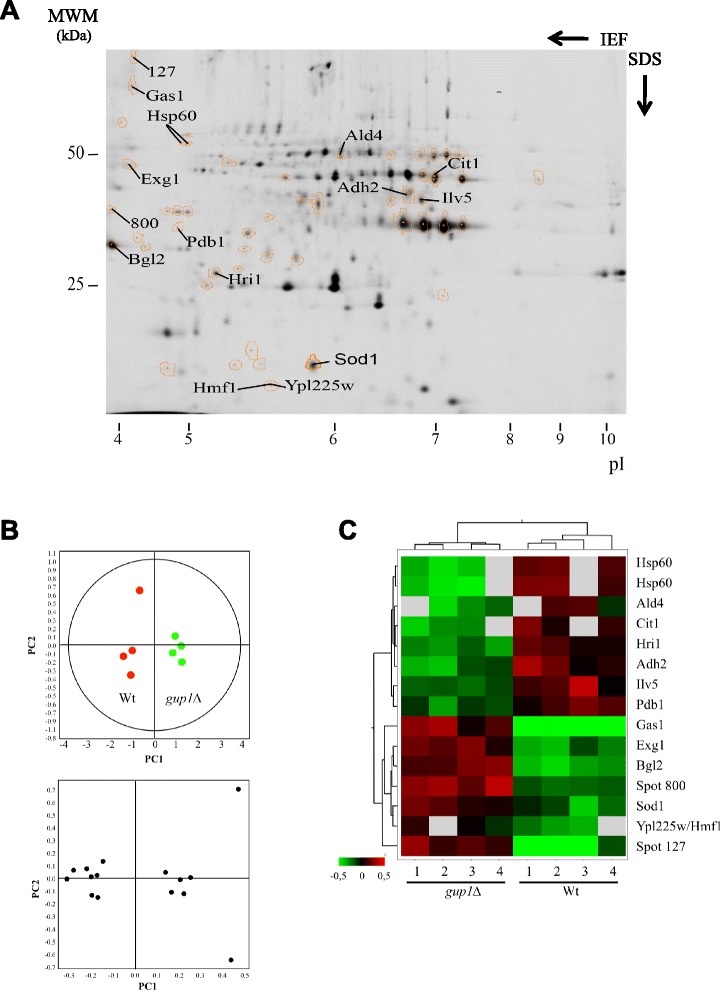
Identification of the yECM proteins changing expression due to the mutation on *GUP1* gene. **a** 2D DIGE analysis of the samples 1 and 2, showing the identification of the protein spots which abundance varied ≥ 2-fold and present *p* < 0.05. **b** and **c** Unsupervised multivariate analysis of data from the 2D DIGE experiment. **b** Upper panel: Principal Component Analysis showing respectively the clustering of the eight individual Cy3- and Cy5-labeled DIGE spot maps; Lower panel: score plots showing the subset of proteins whose ratios varied 2-fold or more and in which *p* < 0.05 in the two principle components. **c** Heat map of the relative protein expression values, each vertical lane corresponding to a sample from A - upper panel, and each horizontal lane a protein spot from A - lower panel. Hierarchical clustering settings are Pearson distance measurements and average linkage. The dendrogram of eight individual spot maps clustering is shown at the top, and that of individual proteins is shown on the left

The proteomic profile of DIGE (Fig. 5a) analysed in the DeCyder Software, revealed an average of 1400 spots per sample, including protein isoforms. The biological variation analysis (not shown) matched a total of 1200 spots that were present in every sample. This spot set was used to study the proteins that are differentially abundant in the yECM of the wt and *gup1Δ* yECM samples. A total of 56 protein spots presented significantly different *p* < 0.05, abundance variation 1.5-fold or greater between the two strains. The mutant strain presented 28 spots with increased abundance, and another 28 spots that were significantly less abundant than in the wt. Among these 56 protein spots, the 15 ones presenting an abundance variation of 2-fold or greater, orange-marked spots in Fig. 5a, were excised from the gel, subjected to tryptic digestion, and the resulting peptides analysed by MALDI-TOF/TOF (Table 2). Eight spots presented ≥2-fold increased abundance in the mutant sample (Table 2 upper half), and another 7 were more abundant in the wt sample (Table 2 lower half). These results were equally obtained in four replicates. All the proteins identified with this procedure were also identified by Nano LC-MS/MS in the yECM from both wt and mutant strains (db PXD001133).

A pattern analysis was performed to assess the differential abundance of proteins in the several replicates and validate the reproducibility of the extraction methodology (Fig. 5b). The Principal Component Analysis displays the two main components that distinguish the two largest sources of variation within the data set. The data sets from four replicates were grouped together with the first component PC1, in the loading plot (Fig. 5b top panel), showing that the samples present a low variance within the same strain. The score plot shows that eight proteins are more present in the wt, while seven are more abundant in the mutant (Fig. 5a, lower panel), accordingly to the results in Table 2. Subsequently, the similarities of protein abundance between samples was analysed through the Hierarchical Clustering Analysis (Fig. 5c). Samples formed two main clusters, one including all the wt replicates and other including all the *gup1Δ* replicates. The variations between each replicate are displayed as an abundance matrix, represented in a standardized logarithmic scale of abundance ranging from −0.5 (green) to +0.5 (red).

#### *Proteins differentially expressed in yECM from wt and* gup1∆ *strains*

The proteins found in higher abundance in the *gup1Δ* mutant in relation to wt Table 2 upper half, were mainly associated with cell wall assembly and maintenance: Gas1 β-1,3 glucanosyltransferase, [67], Exg1 exo-1,3-beta-glucanase, [68], Bgl2 endo-beta-1,3-glucanase, [69], and Sod1 superoxide dismutase, [70]. Gas1 displayed the biggest difference between wt and mutant, the latter presenting ≥8 times more protein than the former. On the other hand, Exg1, Bgl2 and Sod1 were previously described to locate to the cell surface [45, 59, 70], and Exg1 and Bgl2 were respectively reported and informatically predicted to the extracellular region [40, 71]. As suggested before [31], the altered membrane and cell wall composition and structure characteristic of the *gup1∆* could be the cause of releasing from the cell surface proteins weakly attached to the wall. Finally, two uncharacterized proteins, Hmf1 and Ypl225w, were also identified. These two proteins were not sufficiently resolved in the 2DE and were identified from the same spot (Fig. 2a; Table 2), being difficult to realise whether both are actually affected by the *GUP1* deletion. Finally, the protein spots n° 127 and 800 (Table 2) were not identified, as it was not possible to extract enough protein to perform an unequivocal identity assignment.

In opposition, the wt ECM proteins with significantly increased secretion, when compared to the mutant ECM, mostly relate to metabolism and their increased amounts do not go beyond 2–2.5 fold. The exception is Hsp60 chaperone that is up to 4 times more secreted (Table 2, lower half). In particular, two isoforms of Hsp60 were identified that suffered the higher decrease in abundance with the deletion of *GUP1* (−3.73 and −4.38 fold). Hsp60 is a mitochondrial chaperone involved in the *de novo* folding of mitochondrial proteins and respiration complexes assembly [72], and it is a vital protein, since cells lacking the gene encoding for this protein are unviable [73]. The remaining proteins that were significantly less present in the mutant yECM can be ascribed to i) the metabolism of ethanol (Ald4 and Adh2) and Acetyl-CoA (Cit1 and Pdb1), ii) protein folding and targeting (Hri1 besides Hsp60), and iii) maintenance of mitochondrial DNA (Ilv5). Moreover, these results revealed a surprisingly small list of differently expressed yECM-only proteins, *i.e.,* the proteins from yECM that were not found also in LGM (Table 4).

**Table 4:** List of the *S. cerevisiae* ECM-only proteins identified as being differently expressed in wt and *gup1∆* mutant by 2D DIGE. Proteins common to liquid growth medium are not presented

	Characterized ORFs		Uncharacterized ORF/protein w/unknown function
wt ECM > > *gup1∆* ECM	Adh2	Alcohol dehydrogenase II	Hri1
	Ald4	Aldehyde dehydrogenase	
	Cit1	Citrate synthase	
	Pdb1	Pyruvate dehydrogenase (PDH) complex (E1 β subunit)	
*gup1∆* ECM > > wt ECM	Exg1	Exo-1,3-beta-glucanase	YPL225w
	Hmf1	Heat shock protein; member of the p14.5 multifunctional protein family (Marchini et al., 2002)	

Adh1 and Ald4 are the enzymes that catalyse the two consecutive steps needed to convert ethanol into acetaldehyde and acetate, and Pdb1 and Cit1 the enzymes that convert pyruvate into acetyl-coA and citrate. Citric acid is broadly used as cross-linker in bio-fibrous materials development [74]. It reduces moisture absorption, the molecular movement, and swelling at high relative humidity, while reducing gas permeability. All this is achieved at around pH 6, which is a physiological pH for yeast communities. Citrate is also used to cross-link the polysaccharide hyaluronan from mammalian ECM. On the other hand, acetate is used in material sciences as a neutralizer for crosslink stabilization [75]. The presence of these four enzymes in the yECM, and their reduced amounts in the mutant as compared to wt, suggest that citrate and acetate might be regular components of yECM and could be important for texture reinforcement. This concurs with previous data observed in colonies of *S. cerevisiae* [43], and with the many oxidative stress-related proteins found in ECM (not shown, PXD001133).

Hri1 has no known biological function, although it was found to interact with members of the yeast Sec 63 complex used for translocation of pre-secretory proteins into the ER [76]. Hri1 protein was overproduced in yeast in response to the overexpression of human Z-type α1-antitrypsin [77]. This is a mammalian serpin known to negatively regulate neutrophil elastase, an extracellular protease that destroys elastin and other connective tissue matrix structural components [78]. This protein is actually considered a negative regulator of ECM proteases in mammalian cells [79]. Interestingly, Hri1 exists as a homo-dimer that binds TEG and SO_4_ (RCSB Protein Data Bank - 3RBY). The presence of sulphated sugars in the yECM has been previously suggested [80]. A direct or indirect biological role of putative Hri1 dimers as well as citrate and acetate in the physical-chemical integrity of yECM is suggested by the aforementioned *gup1∆*-associated change in ECM structure.

## Conclusions

This work presents a first comprehensive assessment of the proteome from the extracellular matrix of *S. cerevisiae* yECM, grown onto biofilm-like mats. This yeast, in opposition to the human pathogen *C. albicans*, has been poorly assessed in what regards the biology of multi-cellular aggregates (for a review see [81]). Most of the work found in the literature addressed colonies on agar, or consortia from flocculating fermentations. The present work has overcome the limitations inherent to those approaches using biofilm-like mats broad homogenous biomass [37] that allows the application of analytical methods without sample size restrictions. The global picture that emerges is that yECM is a highly complex proteinaceous substance as inferred from the extremely large number and diversity of proteins it harbours. Further considering the large amount of polysaccharides also present in the yECM [80], which full chemical and structural characterization is not yet available, makes of yECM an environment molecularly equivalent to high Eukaryotes extracellular matrix. This is stressed by the fact that many of the proteins identified are common to mammalian or other high eukaryotic ECM.

yECM displays large sets of enzymes from both glycolytic and gluconeogenic pathways, suggesting that *S. cerevisiae* mats, as previously described for this yeast colonies [42, 43] or *C. albicans* biofilms [44], actually harbour two metabolically distinct populations, one feeding on glucose and another on ethanol possibly produced by the first. Many types of microbes including yeasts are well known to secrete enzymes that are active outside the cell, like amylases, esterases, lipases, proteases, pectinases, chitinases and cellulases (e.g. [82, 83]), the most well known being invertase that dissociates sucrose components turning them available for yeast consumption. Furthermore, in more complex multicellular organisms, some metabolic functions actually take place in the periplasmic space (e.g. [84]) or within the ECM (for a review see [23]). Therefore, the presence of complete sets of enzymes from specific metabolic pathways outside the cell may correspond to one of two things: either these enzymes perform a role identical to the one they have inside the cell, and therefore metabolic reactions occur in the extracellular surroundings, or they act as *moonlighters* [85]. This term was created to coin proteins like yeast Tdh3 [86] and Eno1/2 [87], which perform unpredicted roles at unpredicted locations apart from their regular enzyme intracellular assignment in a given metabolic pathway. In particular, Tdh3 was suggested to act as fibronectin and laminin-binding protein [86], mediating yeast adhesion to tissues during pathogenic invasion, consistent with the reported overexpression of this protein in biofilms from *C. albicans* and its implication in their development [12]. On the other hand, Eno1 has a role in cancer aggressiveness, by promoting cell invasion and metastasis through activation of plasminogen into plasmin [41], as well as in rheumatoid arthritis by promoting the migration of fibroblasts [88]. Identically, during *C. albicans* infections, Eno1 binds host cells plasminogen and plasmin therefore facilitating tissue invasion [89], playing a complex role that makes it an immunodominant antigen [87]. Concomitantly, Eno1 has a role in *C. albicans* biofilm development and yeast-to-hyphae dimorphic transition [13]. Besides these two enzymes, Pgk1, Pyk1 and Fba1 were also shown to locate at the *S. cerevisiae* or *C. albicans* cell wall outer surface [12, 59, 90], to be exported to the extracellular space through unconventional secretion in vesicles [40, 56], and now to exist in yECM.

In analogy to metalloproteinases from higher Eukaryotes extracellular matrix, yECM presents candidate proteinases that could functionally fulfil a role in matrix remodelling, the Zn-dependent exopeptidases Lap4, Dug1 and Ecm14, the metalloproteases *zincins* Prd1, Ape2 and Zps1, as well as the HSP70 chaperones Ssa1/2/3/4, Ssb1, Ssc1, Sse1/2 and Kar2. Furthermore, again in analogy to higher Eukaryotes, well-established signalling effectors like Bmh1 and Bmh2, or candidate signalling proteins like Tfp1, Vma1 and/or Vde were found in yECM, suggesting that broad signalling events and molecular cell-to-cell communication could occur through the matrix from yeast multi-cellular aggregates.

The results were expanded by using a mutant strain defective in the yeast orthologue of a negative regulator of the Hedgehog signal from high Eukaryotes, *GUP1.* Many proteins from wt ECM were absent from the mutant ECM. These comprise several functional classes: carbon metabolism, cell rescue and defence, protein fate and cellular organization. In particular, in the mutant ECM no fermentation or gluconeogenic enzymes were found, as well as proteins that regulate this pathway, namely the Fbp1 (fructose-1,6-bisphosphatase) and Pyc2 (pyruvate carboxylase). This suggests that the mutant mats do not harbour metabolically distinct populations. Moreover, the mutant ECM has under-represented the enzymes that allow the production of acetate and citrate, which properties as cross linkers might underlie ECM structure and justify mutant’ sliminess.

The yECM emerges as a dynamic and protein rich environment, with high molecular diversity. The majority of the proteins identified are ascribed to intracellular compartments in databases. In fact, only 15 % from the 693 proteins were already annotated to the cell surface, cell wall and/or plasma membrane, and 3.8 % have no ascribed location. Nevertheless, some of the supposedly intracellular proteins were already reported to appear in the cell surface, namely several of the glycolytic/fermentation enzymes [39, 45]. Actually, the unconventional localization of proteins in yeasts appear to be more common than thought [40], and might correspond to *moonlighting* for their unexpected roles, although this designation has been mainly applied to proteins of higher Eukaryotes [85].

The acknowledgement of the existence of structural organization, and differential expression of genes with concomitant metabolic and morphological specialization across yeast colonies or biofilms, is in accordance with yeasts having complex multicellular behaviour. The existence of an extracellular matrix that, in analogy to the higher Eukaryotes, may operate as scaffold but also actively contribute to this behaviour agrees with the suggested complexity. The present work presents the first identification of the proteome of *S. cerevisiae* ECM, which includes several categories of proteins. Sets of intracellular enzymes covering whole metabolic pathways glycolysis, fermentation and gluconeogenesis through the glyoxylate cycle, were found, including the simultaneous presence of glucose induced and repressed enzymes, suggesting that the yECM might allow active metabolism, while receiving proteins from two distinct metabolically active cell populations. Moreover, multiple chaperones and metalloproteinases, and several broad signalling cross-talkers and putative signalling proteins were identified, which suggests the yECM encompasses remodelling and signalling events, analogously to the biological roles from higher Eukaryotes ECM, possibly controlling the fate of the imbedded cells, their survival, differentiation and spatial distribution.

The comparison between the *S. cerevisiae* wt strain and the mutant defective in the *GUP1* gene, which displays an abnormally loose ECM, showed the profound effect that a single gene mutation can have on the yECM. This suggests that yECM composition is highly dynamic and should vary according to microbial species/strain and type of multicellular aggregate. This work opens a door into the understanding of yeast extracellular matrix as a model of primordial processes associated with multicellular life, taking yeast colonies, biofilms, or other types of such large cellular aggregates, as tissue-like communities.

## Methods

### Yeast strains, media and culture conditions

The *S. cerevisiae* strains W303-1A *MATa leu2-3 leu2-112 ura3-1 trp1-1 his3-11 his3-15 ade2-1 can1-100* [91], and BHY54 isogenic to W303-1A but *gup1::His5* [31], were used. Batch cultures were performed in YPD (1 % yeast extract (w/v); 2 % peptone (w/v); 2 % glucose (w/v); 0.005 % adenine hemisulphate (w/v)) in a 2:1 air:liquid ratio, at 30 °C and 200 rpm orbital shaking. Growth was monitored measuring optical density at 600 nm. The development of a yeast biofilm/mat in solid growth medium for the extraction of yECM was performed as previously described [37, 80]. As inoculum, 1.5 ml from a batch culture at OD_600_ 1 was spread evenly onto Ø90 mm YPD plates, supplemented with 2 % agarose (YPDa - [37]). Four independent experiments, each comprising 150 plates inoculated from one batch culture, were used to obtain the results presented for each strain. Yeast viability was checked assessing membrane integrity by flow cytometry, as described before [92]. Briefly, cells were harvested and added 4 μg/ml propidium iodide (PI, Sigma). After 10 min incubation in the dark at room temperature, the samples were analysed in an Epics® XL™ Beckman Coulter flow cytometer.

### yECM extraction and fractionation

The yECM extraction and protein fraction recovery were performed as previously described [37] from 150 petri dishes/strain. As control, proteins secreted to the medium during liquid growth were also assessed. For this purpose, overnight batch cultures were centrifuged for 10 min at 5,000 rpm, and the supernatant was processed in the same way as yECM samples [37]. The freeze-dried extracts were resuspended in MilliQ water, the minimum necessary to completely solubilize the overlay components. The proteins were precipitated using the chloroform/methanol protocol [38]. The protein pellets were left at room temperature to evaporate the remaining methanol, and resuspended in a DIGE compatible buffer (30 mM Tris; 7 M urea; 2 M thiourea; 2 % CHAPS (w/v); pH 8.9). Protein was quantified with Bio-Rad Protein Assay (Bio-Rad, Richmond, CA, USA) as recommended by the manufacturer.

### SDS-PAGE

One-dimensional electrophoretic separation under denaturing conditions (SDS-PAGE) was carried out in a Mini PROTEAN® 3 Cell apparatus (Bio-Rad), in 1.5 mm thick polyacrylamide gel, with a 4 % stacking gel and a 10 % resolving gel. The sample (5 μg) was brought up to 10 μl with MilliQ water and mixed with 5X Laemmli loading buffer [93]. The mixture was boiled for 5 min and then incubated on ice at least 5 min. The electrophoresis was run at 100 V.

For identification of the proteins, 50 μg of total protein extract was brought up to 40 μl with MilliQ water, mixed with Laemmli loading buffer [93], and the run was performed at low voltage 25 V, until the migration front reached 2–3 mm above the resolving gel. The gel was then stained with Colloidal Coomassie Blue. A large band including all the proteins from each sample was excised and used for the identification procedures.

### DIGE of wt *versus gup1∆*

2D Difference Gel Electrophoresis (DIGE) was performed using four independent cultures of wt and mutant strains, generating eight individual samples. Proteins were labelled with Cy dye derivatives according to manufacturer instructions (GE Healthcare Life Sciences, USA). Briefly, each protein sample (50 μg) was labelled with 400 pmol of Cy dye in 1 μl of anhydrous N,N-dimethylformamide (DMF, Sigma). The mixture was incubated on ice in the dark for 30 min. The reaction was stopped with the addition of lysine (10 mM), followed by a second incubation for 10 min on ice also in the dark. The labelled samples were combined as in Table 1, ensuring dye swaps to avoid dye-dependent differences. Each gel contained a pair of Cy3 and Cy5 labelled samples, corresponding to the wt and *gup1Δ*, and a Cy2 labelled pooled standard, *i.e.,* a mixture of equal amounts of all the samples used for each DIGE experiment. To each mixture, an equal volume of 2X hydration buffer (7 M urea; 2 M thiourea; 4 % CHAPS (w/v); 2 % dithiothreitol (w/v); 4 % pharmalytes (v/v); pH 3–11) was added for the cup loading process.

For 2DE first dimension protein separation, 24 cm IPG strips in the pH range of 3–11 were used. The strips were previously hydrated overnight with 7 M urea, 2 M thiourea, 4 % CHAPS (w/v), 100 mM DeStreak, and 2 % pharmalytes (v/v), at pH 3–11. Isoelectric focusing was performed at 20 °C using the following programme: 120 V for 1 h, 500 V for 2 h, 500–1000 V for 2 h, 1000–5000 V for 6 h, and 5000 V for 10 h. Subsequently, the strips were equilibrated for 12 min in reducing solution (6 M urea; 50 mM Tris–HCl; 30 % glycerol (v/v); 2 % SDS (w/v); 2 % DTT (w/v); pH 6.8) and then for 5 min in alkylating solution (6 M urea; 50 mM Tris–HCl; 30 % glycerol (v/v); 2 % SDS (w/v); 2.5 % iodoacetamide (w/v); pH 6.8). The second-dimension SDS-PAGE was run on homogeneous 10 % T and 2.6 % C polyacrylamide gels casted in low-fluorescent glass plates. Electrophoresis was carried out in the dark at 20 °C, with a potency of 2 W/gel for 18 h, using an Ettan-Dalt six unit (GE Healthcare).

### Image acquisition and DIGE analysis

Proteins were visualized using a Typhoon 9400™ scanner GE Healthcare, with CyDye filters. Cy3, Cy5 and Cy2 image acquisition was done at specific excitation/emission wavelengths, respectively 532 nm/580 nm, 633 nm/670 nm and 488 nm/520 nm, and using 100 μm as pixel size. Image analysis was carried out with DeCyder™ differential analysis software (v6.5, GE Healthcare). The Differential In-gel Analysis (DIA) module was used to assign spot boundaries and to calculate parameters such as normalized spot volumes. Inter-gel variability was corrected using a biological variance analysis (BVA) module. The internal standard image gel with the greatest number of spots was used as a master gel. Comparisons between wt and *gup1Δ* mutant were carried out using average ratio and unpaired Student’s *t*-test. In order to reduce the false positive, False Discovery Rate [94] was applied. Protein spots were considered as differentially present with statistical significance between the extracts under comparison if presenting i) a 1.5-fold difference in the average ratio, and ii) a p value less than 0.05. Principal Component Analysis (PCA) and Hierarchical Cluster Analysis (HCA) were performed using the DeCyder Extended Data Analysis (EDA) module on the group of spots identified as significantly changed. Based on collective comparison of expression patterns from the set of proteins, these multivariate analyses clustered the individual Cy3- and Cy5-labeled samples.

### Colloidal Coomassie blue staining and protein digestion

SDS-PAGE and 2DE gels were both stained with Colloidal Coomassie Blue according to published procedures [95, 96], and scanned with a calibrated densitometer (Bio-Rad; Molecular Imager GS-800). The bands containing total protein extract, as well as the chosen spots from the DIGE, were excised and in-gel digested as described before [45]. Briefly, samples were digested overnight at 37 °C with 12.5 ng/μl and 1 μg/20 μg protein of sequencing grade trypsin (Roche Biochemicals) in 25 mM ammonium bicarbonate (pH 8.5) for spots and bands respectively. After digestion, the supernatant from the excised protein bands was analysed by LC-MS/MS and the spots assessed by MALDI-TOF.

### Protein identification

#### LC-MS/MS

Samples of total protein extract (5 μl in 0.1 % formic acid at a final concentration of 1 μg/μl) were loaded onto a C18-A1 ASY-Column 2 cm pre-column (Thermo Scientific) and then eluted onto a Biosphere C18 column (inner diameter 75 μm, 15 cm long, 3 μm particle size, NanoSeparations). The proteins were separated using a gradient on a nanoEasy HPLC (Proxeon) coupled to a nanoelectrospray ion source (Proxeon) at a flow-rate of 250 nl/min. The mobile phase A consisted of 0.1 % formic acid in 2 % acetonitrile, and mobile phase B of 0.1 % formic acid in 100 % acetonitrile. A solvent gradient was applied for 140 min, ranging from 0 % to 35 % phase B. Mass spectra were acquired on the LTQ-Orbitrap Velos (ThermoScientific) in the positive ion mode. Full-scan MS spectra (m/z 400–1800) were acquired with a target value of 1,000,000, at a resolution of 30,000 (at m/z 400), and the 15 most intense ions were selected for Collision Induced Dissociation (CID) fragmentation in the LTQ with a target value of 10,000 and normalized collision energy of 38 %. Precursor ion-charge state screening, and monoisotopic precursor ion selection, were enabled. Singly charged ions and unassigned charge states were rejected. Dynamic exclusion was enabled with a repeat count of 1 and exclusion duration of 30 ms.

Proteome Discoverer 1.2 with MASCOT 2.3 was used to search in the Uniprot/Swissprot taxonomy *S. cerevisiae* database (7798 sequences). The search parameters used were the following: peptide tolerance - 10 ppm; fragment ion tolerance - 0.8 Da; missed cleavage sites - 2; fixed modification - carbamidomethyl cysteine; and variable modifications - methionine oxidation. Mascot ion score 20 and a 99 % peptide confidence were set as filters.

#### MALDI-TOF/TOF

The supernatants from spots excised from 2DE gels were collected, and 1 μl was spotted onto a MALDI target plate and allowed to air-dry at room temperature. Subsequently, 0.5 μl of α̣-cyano-4-hydroxytranscinnamic acid matrix (3 mg/ml) in 50 % (v/v) ACN (Sigma Aldrich), was added to the dried peptide digest spots, and allowed to air-dry again at room temperature. Analyses were performed in a 4800 Plus MALDI TOF/TOF™ and Proteomics Analyzer (Applied Biosystems, MDS Sciex, Toronto, Canada), using 4000 Series Explorer™v 3.5 software (ABSciex). The instrument was operated in reflector mode, with an accelerating voltage of 20,000 V. All mass spectra were internally calibrated using peptides from the auto-digestion of the trypsin. The MS spectra of all the spotted fractions were acquired in positive reflector mode for peak selection signal to noise ratio >12. The suitable precursors for MS/MS sequencing analysis were selected, and fragmentation was carried out using the CID atmospheric gas, on 1 kV ion reflector mode, and precursor mass windows ±4 Da. The plate model and default calibration were optimized for the MS-MS spectra processing.

The search of peptides was performed in batch mode, using GPS Explorer v3.5 software (ABSciex) MASCOT version 2.3 (www.matrixscience.com), and the NCBInr database (a17919084 sequences; 6150218869 residues). The MASCOT search parameters were: i) species - *S. cerevisiae*, ii) allowed number of missed cleavages – 1, iii) fixed modification - carbamidomethyl cysteine, iv) variable modifications - methionine oxidation, v) peptide tolerance - ±50 ppm for PMF and 80 ppm for MSMS searches, vi) MS/MS tolerance - ±0.3 Da, and vii) peptide charge - +1. In all identified proteins, the probability score was *p* < 0.05, *i.e.,* greater than the one fixed by Mascot as significant.

### Western blot

The Western blot was performed as described before [3]. The rabbit anti-VDE antibody was kindly provided by Professor Yoshi Ohya (University of Tokyo, Japan). The reacting polypeptides were visualized using the peroxidase subtract 3,3’-diaminobenzidine Sigma, UK, and an Image Analysis System ChemiDoc XRS Bio-Rad, Laboratories Inc., with Quantity-One 4.5.0 Software (Bio-Rad, Laboratories Inc.).

### Ethics

There are no ethics concerns involved with the experimental design from this manuscript.

## Abbreviations

ECM: Extracellular matrix
yECM: Yeast extracellular matrix
EPS: Extracellular polymeric substance
MBOAT: Membrane-bound *O*-acyltransferase family
Hh: Hedgehog
HHATL: Hedgehog acyltransferase-like protein
wt: Wild type
LGM: Liquid growth medium
TCP: Total cell proteome
ORF: Open reading frame
OD_600_: Optical density (Absorbance at 600 nm)
CHAPS: 3-[(3-cholamidopropyl)dimethylammonio]-1-propanesulfonate
SDS-PAGE: Sodium dodecyl sulphate polyacrylamide gel electrophoresis
2DE: Two dimension electrophoresis
DIGE: 2D Difference gel electrophoresis
HPLC: High performance liquid chromatography
LC-MS/MS: Liquid chromatography-mass spectrometry
MALDI-TOF/TOF: Matrix-assisted laser desorption ionization, time of flight mass spectrometry
CID: Collision induced dissociation
LTQ: Linear trap quadropole
DIA: Differential in-gel analysis
BVA: Biological variance analysis
PCA: Principal component analysis
HCA: Hierarchical cluster analysis
EDA: DeCyder extended data analysis
